# Direct visualization of carbon black aggregates in nitrile butadiene rubber by THz near-field microscope

**DOI:** 10.1038/s41598-023-34565-2

**Published:** 2023-05-15

**Authors:** Youngil Moon, Haneol Lee, Jaekap Jung, Haewook Han

**Affiliations:** 1 Department of Electrical Engineering, Pohang University of Science and Technology, Pohang, 37673 South Korea; 2 Hydrogen Energy Materials Research Center, Korea Research Institute of Standards and Science, Daejeon, 34113 South Korea

**Keywords:** Techniques and instrumentation, Techniques and instrumentation

## Abstract

The use of filling agents for rubber reinforcement is beneficial in various industrial applications, and several experimental methods have been used to study the effect of fillers on rubber. However, due to the lack of a suitable imaging technique, filler dispersion and distribution in rubber cannot be easily displayed. Thus, we utilize the THz near-field microscope (THz-NFM) to directly visualize the distribution of carbon black (CB) aggregates in nitrile butadiene rubber (NBR). The THz time-domain spectroscopy (THz-TDS) was used to evaluate the optical properties of the NBR specimens. Results revealed significant indices contrast between CB and NBR at the THz regime, which was attributed to the variation in electrical conductivities. The micrographs of NBR in the THz-NFM revealed the distribution of CB aggregates. The area fraction (AF) of the CB aggregates was calculated using a binary thresholding algorithm to compare with the transmission electron microscope method. Both methods yielded comparable AF values, suggesting, for the first time, that CB can be detected in the NBR without preprocessing the specimens.

## Introduction

Nitrile butadiene rubber (NBR) is extensively used as a major component in a wide range of rubber applications, and its reinforcement mechanism and structure have been the subject of several studies^1–3^. However, in order to increase its tensile strength, the abrasion resistance and glass transition temperature of NBR require reinforcement^4–6^ despite its excellent and unique qualities. Currently, carbon black (CB) fillers are frequently used as reinforcement agents in the production of rubber compounds^7–9^ because of their low cost and high degree of dispersibility^10,11^. However, the type, shape, and distribution of the filler significantly affect the physical and mechanical properties of the rubber. This makes it very important to investigate the influence of filler on the related properties of rubber. The properties of reinforced rubber with filling agents have been evaluated using various experimental techniques^12–14^; however, studies on the uniformity and distribution of the filling agents in the rubber matrix are rare due to the limitations of investigation techniques. While the scanning electron microscope (SEM), transmission electron microscope (TEM), and atomic force microscope (AFM) are established techniques for examining the microstructures of the different chemical and biological molecules^15–17^, their ability to detect CB aggregates in rubber compounds is limited. The CB detection using AFM is required highly technical preprocessing step using cryo-ultramicrotomes^18–20^. Similarly, the preparation of flat specimens with thicknesses between 20 nm and 100 nm is necessary for CB detection using TEM, which have more stringent requirements^21,22^. Furthermore, SEM cannot clearly distinguish between CB and rubber matrix because most organic elastomers and CB are mainly composed of carbon atoms^23^. Therefore, research on rubber compounds necessitates the development of instrumentation that can detect and identify CB aggregates without the need for pretreatment. Near-field microscopy (NFM) has been developed to study light-matter interaction beyond the diffraction limit of the electromagnetic (EM) wave. The NFM system typically consist of the combining form of AFM and electromagnetic spectroscopy such as Raman and terahertz etc. When a sharp metallic probe approach very close to the surface of a specimen, the incident EM wave localized near the probe apex because of the lightning rod effects. This localized field caused the near-field interaction between probe and sample surface. As a result, the scattered wave is generated from the near-field region and this light can be collected by a detector at far-field and used to create the 2-D near-field image. Therefore, the NFM can also be applied to study the optical properties beyond the diffraction limit such as calculating the local refractive index and the presence of the surface plasmon effect^24–28^. Similarly, in the THz domain, the THz near-field microscope (THz-NFM) technique is an established method for overcoming the diffraction limit of the THz wave^29–31^.

**Figure 1:**
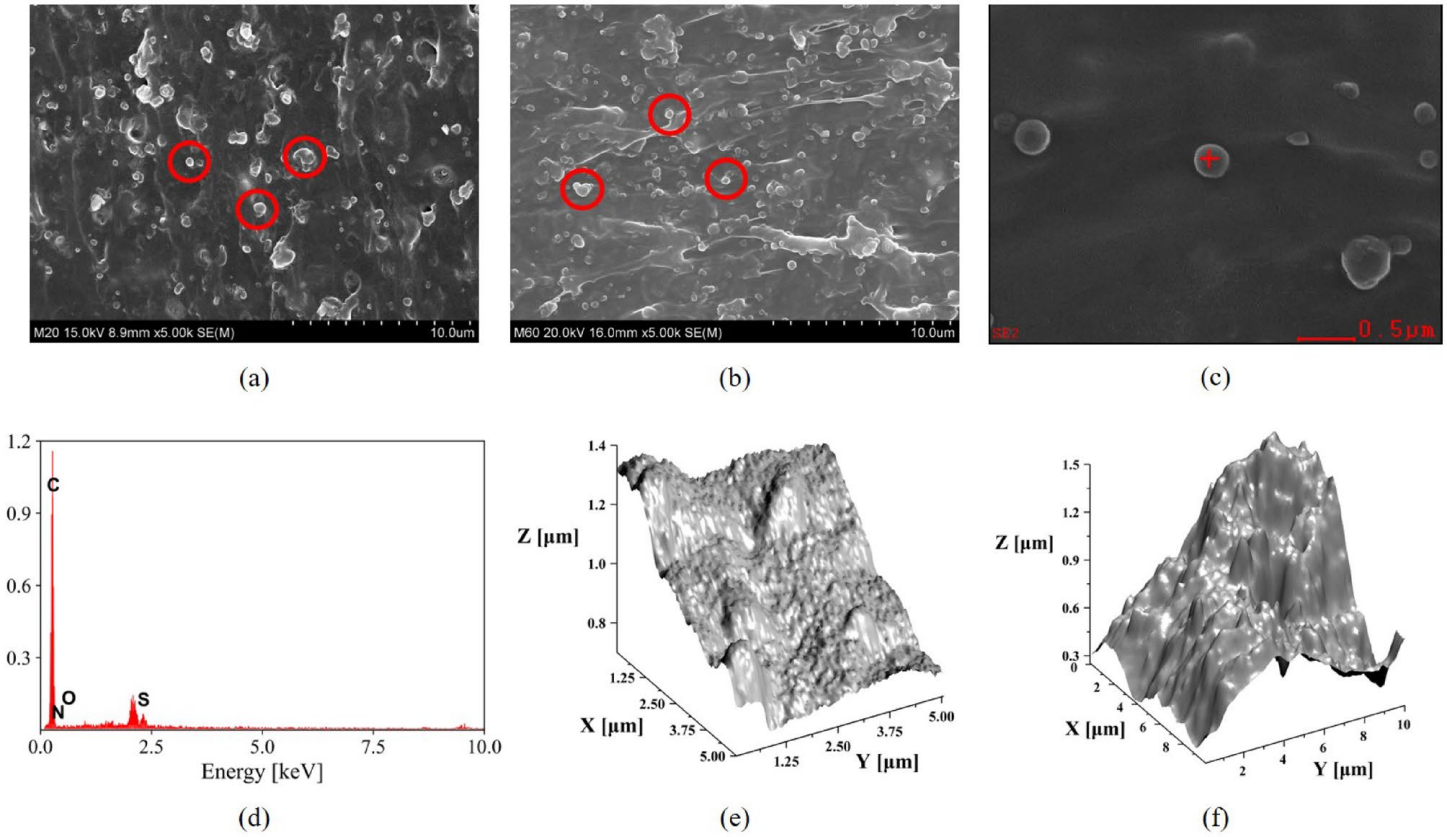
SEM images of NBR M20 (**a**) and M60 (**b**) specimens. Scale bars: 10 $\upmu$m. Grain structures in NBR M60 (**c**); scale bar: 0.5 $\upmu$m. Measured EDS spectrum (**d**) on the nipple structure marked with a red cross in (**c**). AFM 3-D topographic images of NBR M20 (**e**) and M60 (**f**).

Furthermore, the spatial resolution of the conventional THz time-domain spectroscopy (THz-TDS) is limited to a few hundred-micrometer scales^32–35^, whereas the resolution of this THz-NFM has reached 80 nm^36^. Because THz-NFM microstructure imaging is based on the detection of scattered THz near-field intensity, which is highly dependent on the electrical conductivity of the medium under the apex region of the probe, the difference in conductivity between the filling agents and rubber matrix in the THz region should be insured to enable the identification of CB particles, as the mechanism of microstructure imaging is based on this principle. Several studies^37–41^ have investigated the impact of CB fillers on different types of rubber polymers, and their results revealed that CB particles typically have a higher electrical conductivity in the THz domain than the rubber matrix. Therefore, using THz-NFM, we demonstrated the direct visualization of the distributions of CB aggregates in NBR without the need for sample preparation. The advantages and limitations of this method were also elucidated.

## Results and discussion

### CB detection using SEM and AFM

The SEM and AFM were used to investigate the microstructures of CB particles embedded in the NBR. In this study, MT N990 filler was used to compose NBR series. For ease of identification and presentation, NBR blends with fillers were labeled as “NBR-M20” and “NBR-M60”, indicating that NBR contains MT N990 filler in a ratio of 20 or 60 parts per hundred rubber, respectively. The SEM images of NBR M20 and M60 were shown in Fig. 1a, b. The SEM results reveal a few small grain structures which are represented by red circles in Fig. 1a, b. Previous studies have reported that these small grains are CB aggregates^42–44^, however, this information was insufficient to conclude that the grain structures exclusively contained CB fillers. Therefore, EDS analysis was performed on a particular grain to conduct a comprehensive analysis of the elemental composition, as observed in Fig. 1c, d. The grain in NBR M60 (Fig. 1b), with a horizontal dimension of 300 nm, was chosen for the EDS measurement. The EDS spectrum (Fig. 1d) revealed that the grain in NBR M60 carbon was the major element with traces of nitrogen, oxygen, and sulfur atoms, suggesting that the grain structure shown in Fig. 1c is not a CB aggregation. Furthermore, the NBR copolymer consisted of polymerized acrylonitrile monomers, and sulfur was added as a crosslinking agent. Thus, the observed nitrile, oxygen, and sulfur EDS signals originated from the rubber matrix. Generally, SEM images were collected by secondary electrons released by the interaction between primary electrons and atomic structure; as a result, the secondary electrons were highly dependent on the atomic number of the specimens.

**Figure 2:**
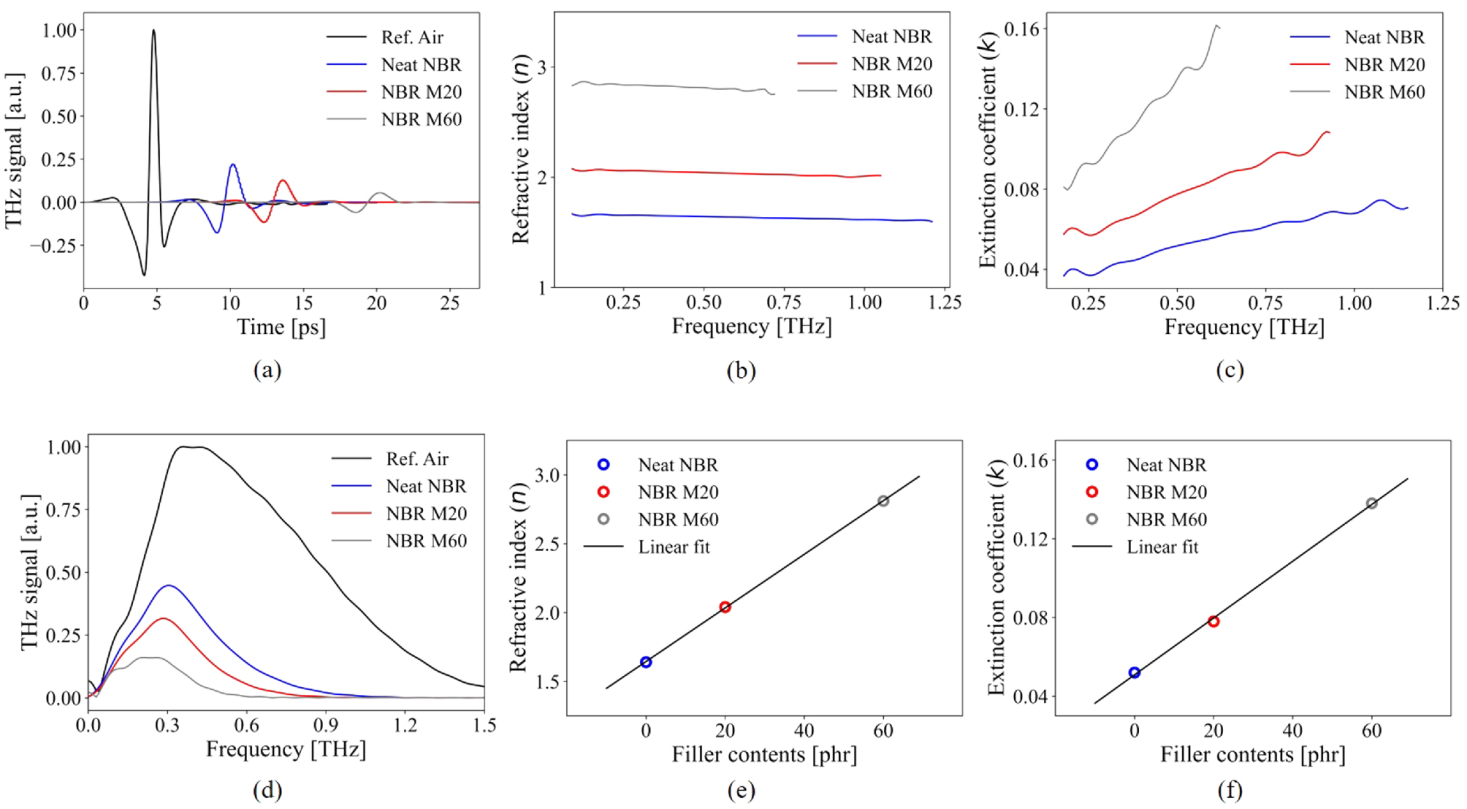
Time-domain (**a**) and frequency domain (**d**) data of neat NBR, NBR M20, and M60. Black solid line; reference signal in air. The blue, red, and gray solid lines indicate the results of neat NBR, NBR M20, and M60 specimens, respectively.The real (**b**) and imaginary (**c**) parts of a complex refractive index for neat NBR and NBR with CB fillers. In addition, the calculation results of AC-conductivity using (**b,c**) were shown in Fig. S1a of the supplementary information. Measured both complex indices at 0.5 THz were summarized in (**e,f**). The blue, red, and gray solid lines and empty circles represent the results of neat NBR, NBR M20, and M60 specimens, respectively. The regression in (**e**) is y $=$ 0.0195x $+$ 1.6443, R$^{2} = 0.9999$ and regression in (**f**) is y $=$ 0.0014x $+$ 0.0509, R$^{2} = 0.9988$. R$^{2}$ is the squared correlation coefficient.

Due to the fact that CB and the majority of organic elastomers were mostly composed of carbon atoms, SEM cannot be used to distinguish between CB fillers and the rubber matrix. The imaging area used for the AFM measurements on the NBR M20 and M60 were 5 $\upmu$m by 5 $\upmu$m and 10 $\upmu$m by 10 $\upmu$m, respectively, with an image resolution of 10,000 pixels displayed in Fig. 1e, f. The specimens were not preprocessed in order to compare with the imaging results of the THz-NFM experiment. The AFM topography showed huge height differences in the overall imaging area, making it difficult to differentiate the exact shape and size distributions of CB particles in the rubber matrix. Although the topography displayed protruded surfaces, they cannot be clearly identified as fillers.

#### Complex refractive indices measurement using THz-TDS

A conventional THz-TDS experiment was performed to measure the complex refractive indices of NBR specimens in the THz frequency range in order to evaluate the electrical conductivity contrast between CB and rubber matrix in the THz domain. The incident THz pulse propagated on a path perpendicular to the surface of the NBR rubber. Comparing the THz transmission signal of the sample, the transmitted signal in dry air was used as a reference signal. The THz pulses of samples showed a time delay from the reference signal in the time-domain results (Fig. 2a) due to differences in optical path lengths. As CB fillers increased, the magnitude of time delay increased, while the amplitude of the transmitted THz pulse decreased. After the THz-TDS measurement, the time-domain was converted to the frequency domain, and the result is presented in Fig. 2d. From the figure, it is clear that as the quantity of CB filler increases, the measured frequency domain spectra show a decrease in amplitude. The complex refractive indices were calculated from the THz-TDS measurement using Fresnel’s equation and the Newton-Raphson iteration method and are presented in Fig. 2b, c, respectively. As the CB content increases, both the refractive index and extinction coefficient gradually increase. The complex refractive indices of the samples were recorded at 0.5 THz to investigate the impact of CB particles on the optical properties of the NBR. The refractive index of the neat NBR was 1.64, while that of M20 and M60 were 2.04 and 2.81 (Fig. 2e), respectively. The extinction coefficient was 0.052 for neat NBR, 0.078 for M20, and 0.138 for M60 (Fig. 2f).

**Figure 3:**
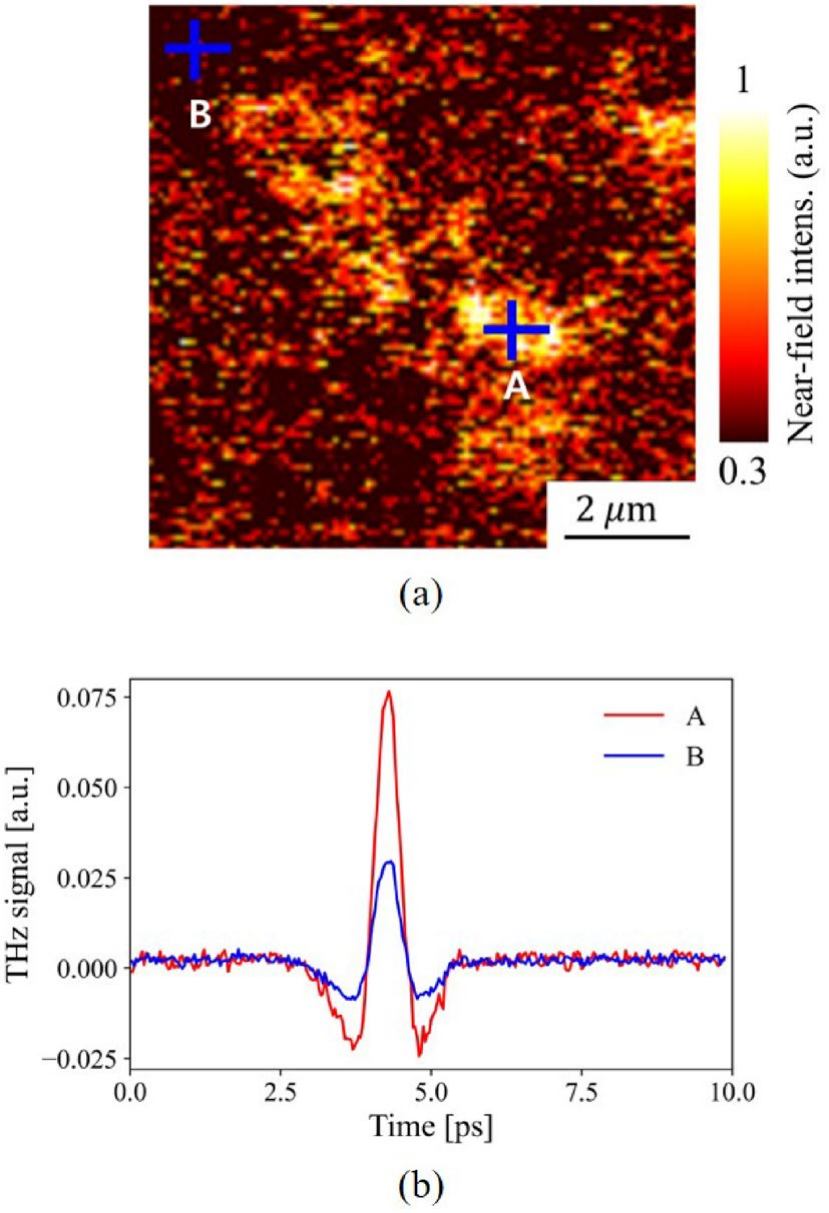
(**a**) THz near field image of NBR M20 and scale bar : 2 $\mu$m. The bright pixels indicate the high intensity of near field signal rather than dark pixel. (**b**) The measured time domain THz near field signals on “A” and “B” point in (**a**). The red and blue solid line indicate the result of “A” and “B”, respectively.

The quantity of CB filler in the rubber matrix showed a linear relationship between the refractive index and extinction coefficient. Furthermore, it can be demonstrated that the complex refractive index and the complex conductivity are theoretically related^45^, and a THz-TDS measurement can be used to determine the relative contrast difference in electrical conductivity between the CB particles and the rubber matrix. Thus, due to the fact that the optical indices of M20 and M60 were greater than the neat NBR, we conclude that the CB particles in the THz domain have higher electrical conductivity in the rubber matrix. In addition, the THz-NFM detects the near-field intensity, which is proportional to the electrical conductivity of under the probe apex region. Thus, based on these spectroscopic results, we anticipate that the THz-NFM would be applicable for direct visualization of CB aggregates in NBR.

#### CB detection using THz-NFM

The optical delay line, which provides a time delay between the probe and pump beam, was used in the THz-NFM measurements to collect near-field THz scattering signals of the NBR samples. The near-field THz images were captured in a 10 $\mu$m by 10 $\mu$m imaging area with a resolution of 10,000 pixel. The optical delay line was fixed at the maximum peak position in the time-domain during the imaging process to ensure a sharp and clear visual contrast between high and low-intensity areas. Since provide the normalized imaging results for neat NBR in Fig. 5, it should normalied with other filler-composited NBR data. Therefore, the near-field THz images(Figs. 3 and 5 ) obtained were normalized by the highest intensity value in the NBR M60 specimen results, which had a higher intensity value than the maximum number in M20. Figure 3a shows the near-field THz image of the NBR M20. The bright pixels in the figure indicate a higher THz scattering intensity than the dark pixels. The near-field THz image of NBR M20 revealed that some regions are clouded with strong near-field interaction between the probe tip and the sample, with a size of  2 $\upmu$m. Furthermore, the marked sections “A” and “B” with a blue cross in Fig. 3a were subjected to time-domain THz near-field spectroscopy by altering the time delay to precisely measure the optical indices of the bright and dark aggregated regions. The apparent signal contrast between “A” and “B” is represented by the detected time-domain near-field signal in Fig. 3b. The maximum intensity of “A” and “B” were 0.077 and 0.03, while the signal-to-noise ratios were recorded at 17.8 and 11.1, respectively.

**Figure 4:**
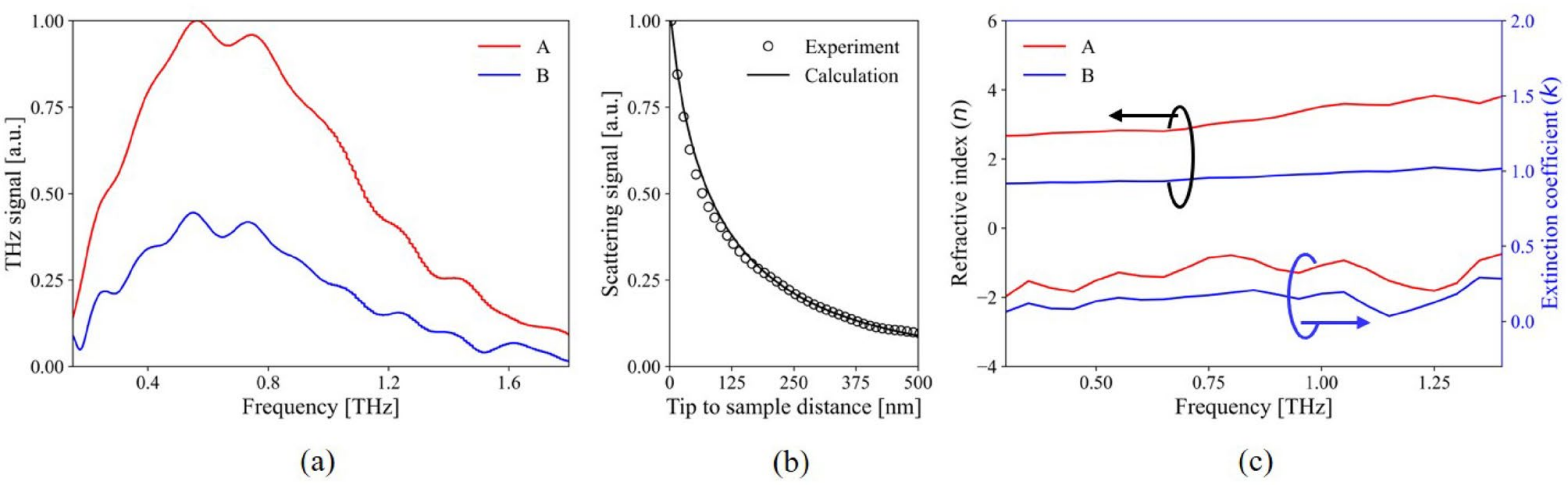
(**a**) Converted near-field signal spectra in the frequency domain of Regions A and B. (**b**) The measured approach curve on a gold substrate is represented as black empty circles. The calculated approach curve of a tungsten probe, which has a radius of 630 nm, is depicted as the black solid line. (**c**) Calculated complex refractive indices of Regions A and B. The red and blue lines in (**a**,**c**) indicate the results of Regions A and B, respectively. Additionally, the calculated AC-conductivity using (**c**) was shown in Fig. S1(b) of the supplementary information.

The measured time-domain signal was converted to the frequency domain, and the converted results are shown in Fig. 4a. The spectra presented in the figure have been normalized using the maximum value obtained from region A (red solid line). The maximum values for regions A and B were 1 and 0.44, respectively. To analyze the THz-NFM spectral results, we developed a self-consistent model based on a quasi-electrostatic image theory called the line dipole image method (LDIM)^29^, which was used to calculate the extinction and refractive indices at the nanometer scale from the frequency domain curve. Fundamentally, the intensity and spectral distribution of the THz near-field signals are highly dependent on the geometric effect of the probe apex, such as its radius. Therefore, geometrically incorrect parameters can lead to miscalculation of the optical constant; therefore, to calibrate the geometry of the probe tip, an approach curve was measured on a gold surface. The curvature and the shape of the approach curve on a gold target enabled the determination of the radius of the probe tip (630 nm), as shown in Fig. 4b. The complex refractive indices in both regions were then iterated using the data from the tip geometry and the converted frequency domain data. The estimated results in Fig. 4c clearly show that in the entire frequency range, both optical indices of region A were greater than the values of region B. Furthermore, the extinction coefficients were measured to be 0.27 and 0.13 for regions A and B, respectively, while refractive indices were determined to be 2.8 and 1.3 for both regions at 0.5 THz. Thus, it is worth noting that the high-intensity signals in the THz-NFM image were predominantly produced by the CB aggregates in the rubber matrix, indicating that the high index of the object medium has higher electric conductivity than the lower index, and based on this analysis, the bright (A) and dark (B) regions of the THz-NFM image are identified as CB aggregates and rubber matrix, respectively.

**Figure 5:**
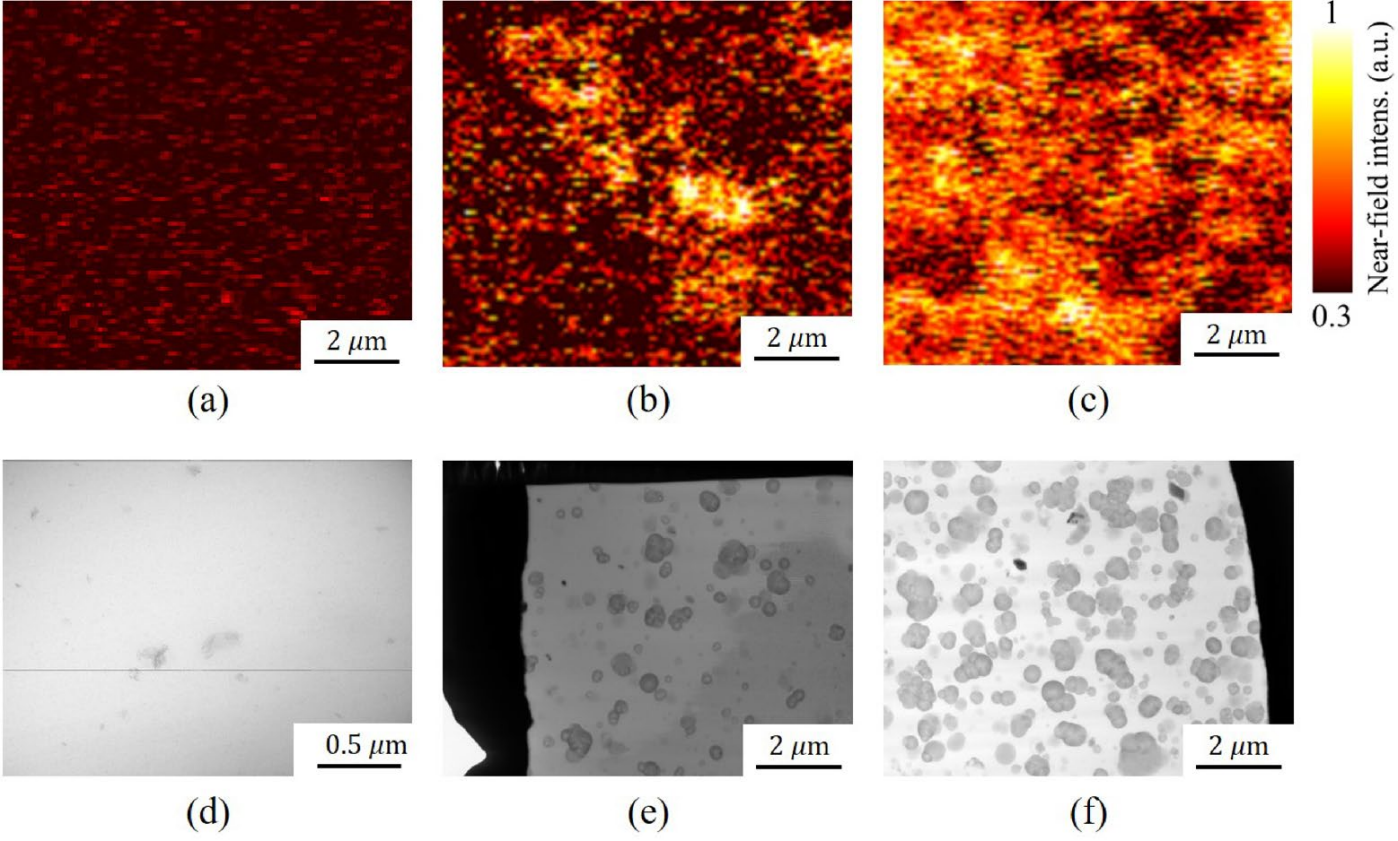
The near-field THz and TEM images of neat NBR (**a,d**), NBR M20 (**b,e**), and NBR M60 (**c,f**). In contrast, the dark aggregations in TEM results represent the carbon black particles, while the bright parts indicate the rubber matrix.

**Figure 6:**
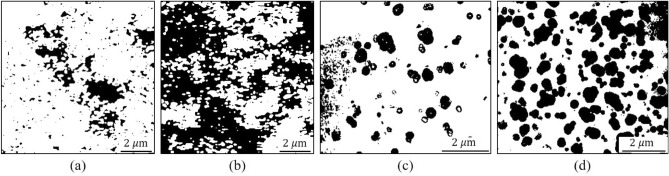
The binarized results of NBR M20 (**a**) and M60 (**b**) by THz-NFM and the images of NBR M20 (**c,d**) for TEM and scale bar: 2 $\upmu$m. The threshold value was set as 200 for all input data. The CB fillers and rubber matrix are depicted as black and white pixels, respectively.

#### Comparison between THz-NFM and TEM techiques

The images in Fig. 5a–c show the THz near-field micrographs of neat NBR, NBR M20, and NBR M60 samples. It is observed that the near-field THz images of NBR M20 and 60 show the presence of CB aggregates with high near-field intensity; the neat NBR exhibits stationary signals in the entire imaging region. As the filler content increased, the number of CB-aggregated areas gradually increased, and we were able to confirm that the CB-aggregated regions were distributed differently in NBR M20 compared to NBR M60. In addition, observations of CB aggregates with the TEM techniques were conducted to compare with the imaging results of THz-NFM. The TEM micrographs in Fig. 5d–f reveal that CB is detected as a dark color. The result of TEM clearly revealed distributions of aggregated and primary CB particles in the rubber matrix, which are similar to the results obtained from the THz-NFM. Thus, we conclude that the CB particles are more densely distributed in M60 than in M20. However, we noticed a disadvantage of the THz-NFM technique: the boundaries of CB particles in the TEM images were easily identified compared to the results obtained from THz-NFM. In the TEM measurement, all specimens were thin films with a thickness of 250 nm; thus, only one layer of CB distributions can exist. Taking into account of the primary particle size of MT N990 CB filler (about 280 nm)^46^, we anticipate the superposition effect of vertical and horizontal directions of CB particles as one of the possible reasons for ambiguity in the images of THz-NFM.

Furthermore, to quantify the dispersity of CB, the area fractions (AF) of CB on both THz-NFM and TEM images were calculated using binary image processing based on “Python.” The calculated AF of CB and binarized image are shown in Table 1 and Fig. 6a–d, respectively. The “OpenCV” module binary threshold method was selected to produce the binarized images. To apply the binary thresholding on an image, a grayscale image is required as an input. Thus, to provide input for binary thresholding, all treated images were first converted to grayscale before covering the black masks on CB particles with the Hue Saturation Value (HSV) color scheme. In Fig. 6a–d, the CB particles and rubber matrix are depicted as black and white pixels, respectively. Fig. 6a, b show the binarized results of NBR M20 and M60 measured by THz-NFM, while Fig. 6c,d show the results of M20 and M60 measured by TEM, respectively. The calculated AF of M20 for NFM and TEM measurements were 13.31% and 14.19%, while the AF of M60 for NFM and TEM measurements were 53.47% and 40.23%, respectively. Although, there was a significant difference in the area fraction for NBR-M60 between the THz-NFM and TEM results, the obtained area fraction for M60 falls within the expected range of uncertainty for both experimental techniques. Therefore, the AF values of CB obtained with both techniques yielded comparable numerical values for M20 and M60 specimens.

**Table 1:** The number of pixels and calculated area fraction results for carbon black (CB) in the rubber matrix are provided, with the Type A uncertainty values shown in parentheses.

	THz-M20	THz-M60	TEM-M20	TEM-M60
Total	151,710	152,100	88,234	87,920
White	131,511	70,619	75,715	52,546
Black	20,199	81,481	12,519	35,374
Area fraction	13.3 (7.5)%	53.6 (6.4)%	14.2 (2)%	40.2 (7.7)%

The uncertainty values were determined using area fraction data from another piece of NBR specimens, and additional experimental results are presented in Fig. S2 and Table S1 of the supplementary information.

## Conclusion

The THz-NFM approach was used to investigate the distributions of CB aggregates in NBR rubber. SEM and AFM measurements were first performed to observe the CB aggregates; however, when compared to the THz-NFM measurements, the obtained images did not show a distribution of the CB aggregates. The complex refractive indices of NBR specimens were determined using THz-TDS to evaluate the electrical conductivity contrast between CB fillers and rubber matrix at the THz region. The results indicated that the electrical conductivity of NBR specimens increased as CB fillers increased. Therefore, a CB detection experiment using THz-NFM was conducted, and the measured near-field maps for NBR specimens revealed clear and distinct CB aggregate distributions in the rubber matrix. Furthermore, the real and imaginary parts of complex refractive indices on CB aggregates were precisely measured to be 2.8 and 0.27 at 0.5 THz, respectively, and the corresponding indices on the pure rubber region were 1.3 and 0.13, respectively. The time-domain near-field spectroscopy at the nanometer scale was also performed on CB aggregated and pure rubber regions. Lastly, TEM measurement was performed to compare with the imaging results of THz-NFM. The results of TEM revealed distinct distributions of CB aggregates in rubber compared to THz-NFM, and we believe that this is caused by the superposition effect of surrounding CB particles along the surface area. Furthermore, when binary threshold image processing was used to calculate the area fraction of CB in both methods, the results revealed comparable numerical values. To the best of our knowledge, this is the first time that the distributions of CB aggregates in rubber have been detected and measured without the specimen being preprocessed. Therefore, we believe that the THz-NFM method can enable a simple imaging method to investigate the microstructures of various polymer composites in the THz region without the need for additional sample preparation.

## Methods

### Nitrile butadiene rubber composite

**Figure 7:**
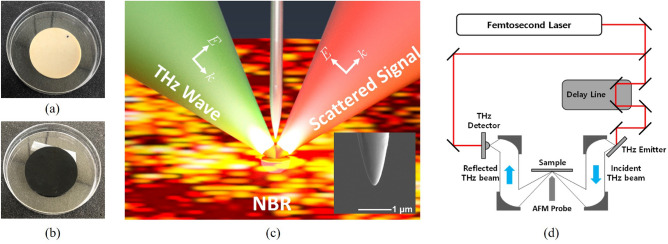
The photographs of neat NBR (**a**) and NBR M20 (**b**) specimens. (**c**) The schematic of THz-NFM, the inset in (**c**) represent the SEM image of the homemade tungsten tip and scale bar: 1 $\upmu$m. (**d**) Detailed description of our THz-NFM set-up.

The Kumho petrochemical group’s KNB 35 L (Kumho NBR), with an acrylonitrile concentration of 34 wt.%, was used as the main component to produce the neat NBR rubber shown in Fig. 7a, the detail chemical compositions are shown in Table 2. CB fillers with particle sizes ranging from 250 nm to 350 nm were used in this study. For every hundred pieces of rubber, the vulcanizates were loaded with either 20 or 60 parts of rubber. For easy identification and presentation, the NBR blends with fillers were labeled “NBR-Mx”, indicating that NBR contains CB. The synthesized NBR sheets were cut with a round hole punch, and the prepared NBR specimens were shaped into cylindrical forms with a diameter of 50 mm and a thickness of 2 mm (Fig. 7a, b).

### SEM, TEM and AFM measurements

The performance of the THz-NFM technique for microstructure imaging was compared using SEM, TEM, and AFM measurements. The SEM and energy dispersive X-ray spectroscopy (EDS) were carried out on a Hitachi S-4700 SEM (Tokyo, Japan) equipped with an energy dispersive X-ray spectrometer (EDS, EMAX energy EX-200, Horiba, Kyoto, Japan). Before conducting the SEM and EDS experiments, Hitachi E-1030 ion sputtering (Tokyo, Japan) was used to coat the surface of the NBR samples with platinum to reduce thermal damage and improve the secondary electron signal^47^.

**Table 2:** Chemical composition of the neat NBR and NBR with N990 CB fillers.

Chemical composition	Neat NBR	NBR M20	NBR M60
KNB 35L	100	100	100
ZnO	3.0	3.0	3.0
St/A	1.0	1.0	1.0
MT N990	–	20	60
S	1.5	1.5	1.5
TBBS	0.7	0.7	0.7

The accelerating voltage for NBR M20 and NBR M60 was 15 kV and 20 kV, respectively. TEM observations were conducted using a TECHNAI F20 (FEI, USA) instrument with an accelerating voltage of 200 kV. Before evaluating the microstructure of CB using TEM, all specimens were preprocessed with the focused ion beam technique to create a thin foil specimen with a thickness of 250 nm. The AFM experiments were conducted in a noncontact mode custom-built system. The AFM tip’s dithering frequency was measured to be 27 kHz. The dithering amplitude and the tip-to-sample distance were precisely controlled during the measurement, using proportional-integral-derivative control.

### THz time-domain spectroscopy

THz-TDS measurements were conducted to evaluate the electric conductivity of NBR specimens in the THz frequency range. To operate the THz-TDS system, a source with a pulse width <100 fs was used with an 800-nm femtosecond laser as a source beam. Semiconducting wafers made of InAs and LT-GaAs were used as a THz emitter and detector, respectively. The THz-TDS experiments were all performed at 300 K. To avoid water absorption on a THz wave, the THz-TDS system was enclosed in a dry air filled acryl chamber capable of maintaining a dew point below $-45{} \, ^{\circ }\hbox {C}$ during the experiment.

### THz near-field microscopy

The distributions of the CB aggregates in NBR specimens were observed using a custom-made THz-NFM system. Fig. 7 shows the THz-NFM schematic with two NBR specimens. A tungsten wire with a diameter of 50 $\mu$m was used as the probe tip, and an electrochemical etching procedure created the probe (inset in Fig. 7c). The W wire was immersed in a 1-N NaOH solution and biased by 4 V from a voltage source. The over-etching time (ts) after reaching a current that cut through the wire was used to determine the size of the probe tips. A quartz tuning fork was fitted with the fabricated probes. The THz pulses generated by the InAs THz emitter were collimated and focused using gold-coated parabolic mirrors. The tungsten probe’s THz near-field scattering was focused on a photoconductive antenna in the far-field region, where the incident and scattering angles were both 60$^{\circ }$, as shown in Fig. 7c, d. The first harmonic of the photocurrent in the THz antenna was measured using a lock-in amplifier to demodulate the probe-dithering frequency of the quartz tuning fork. The detection lock-in time constant was set to 300 ms, and the detected time-varying THz scattering signals were recorded using a custom-made measuring software developed with “LabVIEW”.

## Supplementary Information

Supplementary Information.

## Data Availability

The datasets generated and/or analyzed during the current study are available from the corresponding author on reasonable request.
