# Author Correction: Direct visualization of carbon black aggregates in nitrile butadiene rubber by THz near-field microscope

**DOI:** 10.1038/s41598-023-35961-4

**Published:** 2023-06-01

**Authors:** Youngil Moon, Haneol Lee, Jaekap Jung, Haewook Han

**Affiliations:** 1 Department of Electrical Engineering, Pohang University of Science and Technology, Pohang, 37673 South Korea; 2 Hydrogen Energy Materials Research Center, Korea Research Institute of Standards and Science, Daejeon, 34113 South Korea

Correction to: *Scientific Reports* 10.1038/s41598-023-34565-2, published online 15 May 2023

In the original version of this Article Haewook Han was incorrectly affiliated with ‘Hydrogen Energy Materials Research Center, Korea Research Institute of Standards and Science, Daejeon 34113, South Korea’. The correct affiliation is listed below.

Department of Electrical Engineering, Pohang University of Science and Technology, Pohang 37673, South Korea.

The original Article has been corrected.

