# Supplementary material for: Direct visualization of carbon black aggregates in nitrile butadiene rubber by THz near-field microscope
Source: Sci Rep. 2023 May 15;13:7846. doi: 10.1038/s41598-023-34565-2 (PMC10185497; doi:10.1038/s41598-023-34565-2)
Supplement: Supplementary file 1 — Supplementary Information. [file 41598_2023_34565_MOESM1_ESM.docx]

**Supplementary Information**

Direct Visualization of Carbon Black Aggregates in Nitrile Butadiene Rubber by THz Near-field Microscope

Youngil Moon^1^, Haneol Lee^1^, Jaekap Jung^2,*^, and Haewook Han^1,**^

^1^Department of Electrical Engineering, Pohang University of Science and Technology, Pohang, 37673, South Korea

^2^Hydrogen Energy Materials Research Center, Korea Research Institute of Standards and Science, Daejeon 34113, South Korea

*jkjung@kriss.re.kr

**hhan@postech.ac.kr

The calculation of ac-conductivity (as shown in Fig. S1) at THz frequency domain for the obtained results of Fig. 2b, c and Fig. 4c has been conducted with using the following relation (eq.1 and 2) between complex refractive index and conductivity [1].

|  | $\left( n+i\kappa\right)^{2}=\varepsilon^{'}+i\varepsilon^{"}$ | (1) |
| --- | --- | --- |

Where, $\varepsilon^{'}$ and $\varepsilon^{"}$ represent the real and imaginary part of the complex permittivity. The dielectric loss ($\varepsilon^{"}$) can be represented by the $\varepsilon^{"}=\frac{\sigma_{ac}}{\varepsilon_{0}\omega}$, where $\sigma_{ac}$ is the AC-conductivity, $\varepsilon_{0}$ is the permittivity of the free space, and $\omega$ is the angular frequency. Consequently, the refractive index can be expressed in terms of conductivity as follow:

|  | $\sigma_{ac}=2\varepsilon_{0}\omega n\kappa$ | (2) |
| --- | --- | --- |

Fig. S1(a) shows the AC-conductivity of the NBR specimens obtained by THz-TDS. The results indicate that the conductivity of NBR gradually increases with the amount of CB filler at the THz frequency range. Fig. S2(b) displays the results of the THz-NFM experiment at positions 'A' and 'B' in Fig. 3 of the manuscript. As expected, the regions where CB is aggregated exhibit higher electrical conductivity values than the pure rubber regions.

| 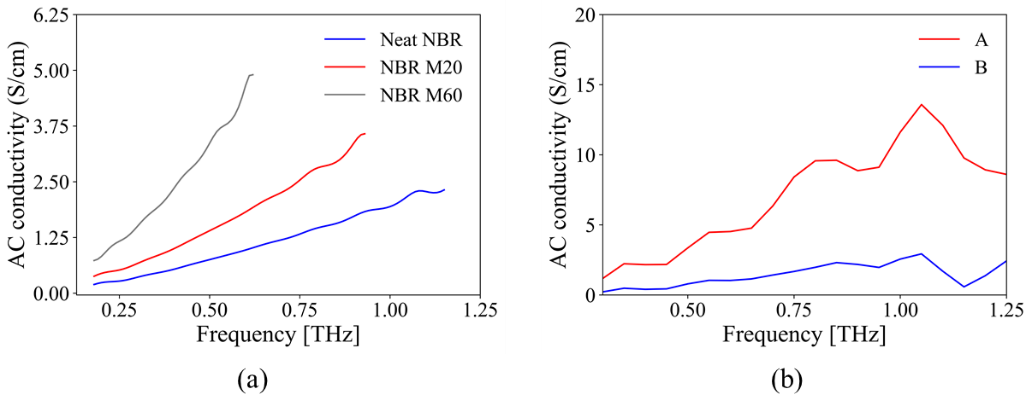 |
| --- |
| Figure S1. Calculated AC-conductivity or Fig. 2 (THz-TDS) and Fig. 4 (THz-NFM) in the manuscript. (a) The calculated AC-conductivity results for THz-TDS experiment. The gray, red and blue solid line accordingly represent the results of NBR M60, 20 and Neat NBR. (b) The calculated ac-conductivity for THz-NFM experiment. The red and blue solid line represent the results on the position A and B in Fig. 3. |

We also investigated the AF value of the CB aggregates in NBR using THz-NFM and TEM, by examining different positions of the NBR pieces (see Fig. S2 and Table S1). The imaging results in Fig. S2 were obtained from an area of the 5 $\mu m$ by 5 $\mu m$. The results of THz-NFM have the imaging resolution of the 2500 pixel, and the presented intensities are normalized by the highest intensity value of the NBR-M60 in Fig. 5c. Using these results, Type A uncertainties are calculated for the results presented in Table 1 of the manuscript.

| 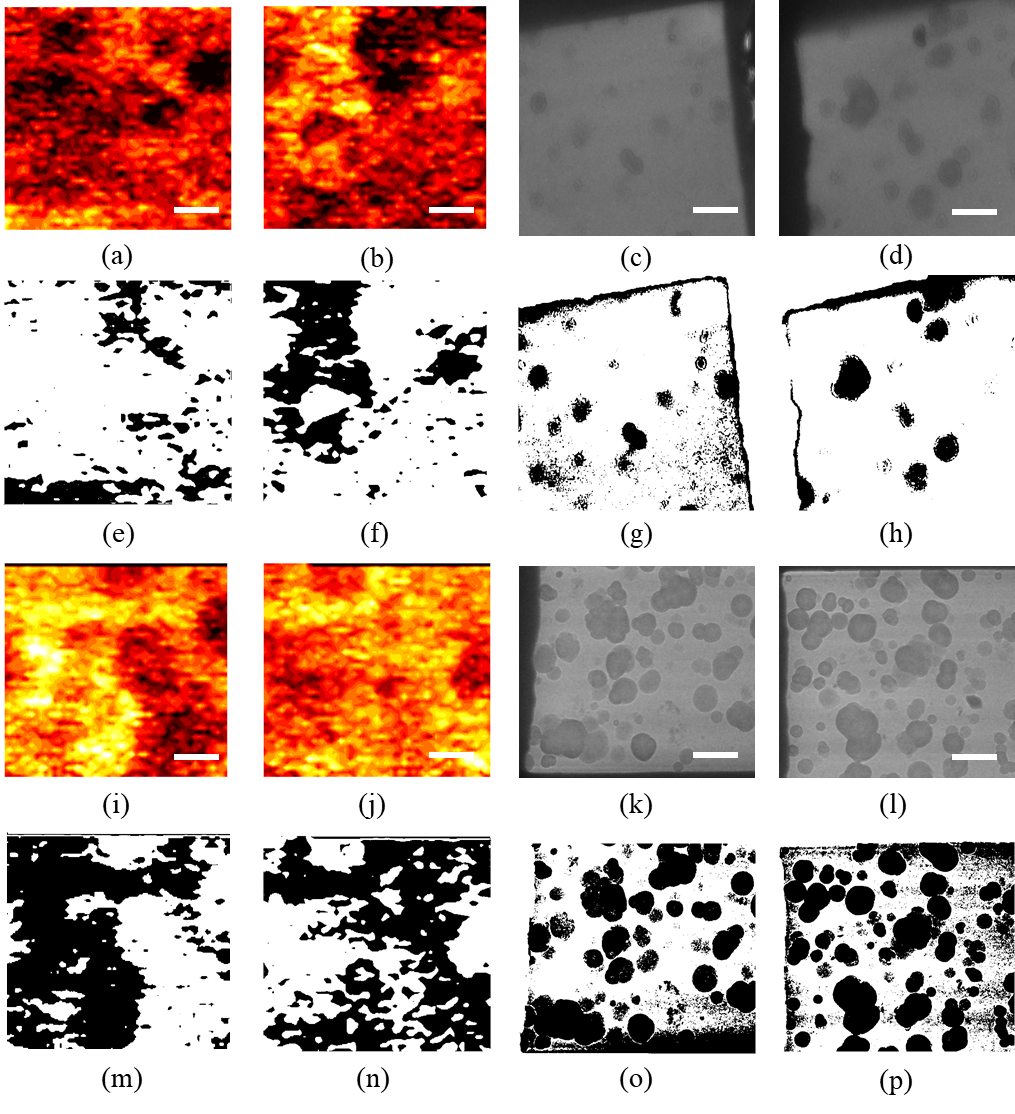 |
| --- |
| Figure S2. (a, b) and (i, j) respectively represent the imaging results for the NBR-M20 and 60 by THz-NFM method. While (c, d) and (k, l) represent the imaging results for the NBR-M20 and 60 by TEM method, respectively. Scale bar: 1$\mu m$. (e-h) and (m-p) show the binarized images for the NBRM-20 and 60 results in (a-d) and (i-l), respectively. |

| Figure for AF calculation | Area Fraction (%) |
| --- | --- |
| e (NBR-M20 by THz-NFM) | 17.6 |
| f (NBR-M20 by THz-NFM) | 20.8 |
| g (NBR-M20 by TEM) | 14.4 |
| h (NBR-M20 by TEM) | 12.2 |
| m (NBR-M20 by THz-NFM) | 47.2 |
| n (NBR-M20 by THz-NFM) | 54.4 |
| o (NBR-M20 by TEM) | 43.6 |
| p (NBR-M20 by TEM2) | 47.9 |

Table S1. The calculated area fraction for CB in rubber matrix with different imaging position and method.
